# Endodontic Treatment of a Double-Rooted Maxillary Second Molar with Four Canals: A Case Report 

**Published:** 2014-10-07

**Authors:** Hengameh Ashraf, Omid Dianat, Reihaneh Hajrezai, Payam Paymanpour, Sina Azadnia

**Affiliations:** a *Iranian Center for Endodontic Research, Department of Endodontics, Dental School, Shahid Beheshti University of Medical Sciences, Tehran, Iran;*; b *Department of Orthodontics, Dental Branch, Islamic Azad University, Tehran, Iran;*; c *Department of Endodontics, Dental School, Shahid Beheshti University of Medical Sciences, Tehran, Iran; *

**Keywords:** Cone-Beam Computed Tomography, CBCT, Maxillary Second Molar, Root Canal Therapy, Tooth Root

## Abstract

A healthy female was referred to Endodontic Department. The referral letter from her dentist expressed that an emergency pulpotomy of tooth #27 had been carried out with probable perforation of the chamber floor which was due to the unusual anatomy of the chamber. Cone-beam computed tomography (CBCT) revealed that the tooth had two mesial and two distal canals. Perforation site was repaired and endodontic treatment was completed. At 24-month follow-up, patient was asymptomatic and clinical and radiographic examinations showed successful outcomes.

## Introduction

Knowledge about the anatomical variations of the tooth morphology and pulp chamber is a prerequisite for successful endodontic treatment. Inadequate treatment of all root canal system can lead to failure of treatment [1, 2] . Therefore, a comprehensive knowledge about anatomic and morphological variations of the root canal system is very important [3] . From the anatomical point of view, permanent maxillary second molars (MSM) often have three roots including one palatal (P) and two buccal (B) roots each containing one canal. Presence of a second canal in the mesiobuccal (MB) root is common, though not as common as its prevalence in maxillary first molars (MFM). If a second canal is suspected, it is barely detectable on routine radiographs [4].

Several studies have reported variations in the anatomy of maxillary molars. Beatty [5] reported a MFM with five canals. Shakouie *et al.* [6] reported three MFMs with fusion of the two buccal roots. Wong described another MFM with the trifurcation of the palatal canal at the apical one third of the root and three separate foramina [7]. Studies have mainly focused on bi- or tri-furcated palatal canals with separate apical foramina [8-11]. Christie and Peikoff [10] evaluated 520 endodontically treated MSM to determine variations in their number of roots and canals. They reported a 1.4% prevalence of two P roots with 4 canals. In addition Shojaeian *et al*. [12] described endodontic treatment of a four rooted MSM with two P canals and a furcal enamel pearl. In another case report by Asgary [13], endodontic treatment of a MSM fused with a supernumerary tooth was discussed. 

However, literature search by the authors yielded no results indicating a MSM with root anatomy similar to that of a mandibular first molar with two M and two distal (D) canals.

## Case Report

**Figure 1:**
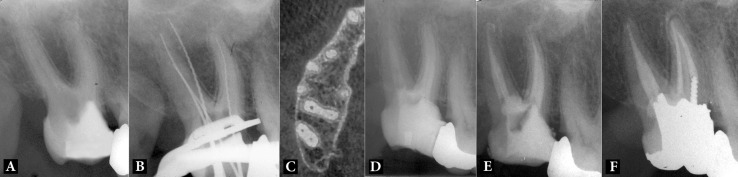
*A*) Pre-operative radiograph, *B*) Perforation of the chamber floor was confirmed, *C*) CBCT of the respective area, *D*) Repair of the perforation site and intra-canal calcium hydroxide medicament, *E*) Post obturation radiography showing four canals, *F*) One-year follow-up

A 57-year-old female with a clear medical history was referred to the Endodontic Department of Shahid Beheshti Dental School (SBDS) by a general practitioner for endodontic treatment of her right MSM. The dentist had performed emergency pulpotomy because of patient’s spontaneous nocturnal pain. The referral letter mentioned unusual anatomy of the root canal system and a probable perforation in the chamber floor during troughing the floor to find the P canal. Initial pretreatment panoramic radiography revealed an extensive carious lesion at the mesial surface of the right MSM and root canal calcification. After taking periapical radiograph, a long distal root was evident but the palatal root was not clear; which is not uncommon considering the possibility of superimposition of P and DB roots. Except for a slight PDL widening, no radiolucency or lesion was observed at the periradicular region (Figure 1A). On clinical examination, the tooth had a temporary coronal filling and was slightly sensitive to percussion and palpation. Periodontal probing was within normal limits. After injection of 2% lidocaine containing 1:80000 epinephrine (Darupakhsh, Tehran, Iran) and placement of rubber dam, temporary filling was removed and the access cavity was reshaped. All orifices were localized; working length (WL) was determined with an electronic apex locator (EAL) (Raypex 5, VDW, Munich, Germany) and confirmed with radiograph. Perforation of the palatal side of the chamber floor was confirmed by means of the EAL and radiographic examination (Figure 1B). The perforation site was temporarily sealed; cleaning and shaping of root canals was performed with ProTaper rotary instruments (Dentsply Maillefer, Ballaigues, Switzerland). During instrumentation, 2.5% sodium hypochlorite (NaOCl) was used as an irrigant. Considering the unusual anatomy of the tooth, orifices were covered with cotton pellets and coronal access cavity was sealed with temporary restoration and the patient was referred to the Oral and Maxillofacial Radiology Department of SBDS to obtain a cone-beam computed tomography (CBCT) scan. The images confirmed the presence of four canals, *i.e* two M and two D canals, within two roots (Figure 1C).

Following ultrasonic debridement and disinfection of the perforation site with 2.5% NaOCl, the area was repaired with Angelus MTA (Angelus, Londrina, Paraná, Brazil). To prevent canal obstruction, orifices were covered with cotton pellets before the application of MTA. Afterwards, intracanal calcium hydroxide (CH) medicament was placed into the canals, a moist cotton pellet was placed over the MTA bulk and the tooth was temporarily restored (Figure 1D).

One week later, after local anesthesia and rubber dam tooth isolation, temporary filling was removed and MTA setting was evaluated. Intracanal CH was washed away with normal saline and 5.25% NaOCl. The canals were dried with sterile paper points (Ariadent, Tehran, Iran) and obturated with gutta-percha (Ariadent, Tehran, Iran) and AH-26 sealer (Dentsply, Tulsa Dental, Tulsa, OK, USA) using lateral compaction technique. Sterile cotton pellets were placed over the orifices and tooth crown was temporarily restored (Figure 1E).

The patient was referred to the Department of Operative Dentistry for permanent tooth restoration. She was put on a regular follow-up and after 12 months, radiographic examination revealed normal periradicular tissues and an asymptomatic and functional tooth (Figure 1F).

## Discussion

This case report presented the endodontic treatment of a double-rooted MSM with two roots that had an iatrogenic perforation on the chamber floor. Two points are noteworthy: the presence of only two roots (absence of P root) and the presence of two canals in each root.

The majority of morphological studies describe the MSMs with three roots and three to four root canals [12]. In the present case, the initial radiograph showed only two roots located at the M and D aspects. In cases where the P root is not evident on the periapical radiograph, the missing P root or fusion of roots are likely.

Presence of two roots in MSM is a rare occurrence. Hartwell and Bellizzi [14] reported only 0.5% occurrence (3 among 538 endodontically treated teeth).

According to Weine *et al.* [15], there may be several types of root canal configuration; a single canal ending with single apical foramen (Type I), two canals joining at the apical region and ending in one apical foramen (Type II), two canals with two separate apical foramina appearing on the root surface (Type III), or one canal bifurcating inside the root and exiting in two separate apical foramina (Type IV). In the present case, both roots were Type II.

Knowledge about the common anatomical shapes and possible morphological variations of the root canal system is critically important in the success of root canal therapy and preventing procedural mishaps. Moreover, when considering the possibility of rare anatomical deviations, evaluation of the radiographs taken with different angles and meticulous assessment of the pulp chamber are critical for adequate endodontic treatment of the root canals and possible accessory canals. In cases where the roots or apices of maxillary molars are not clear on diagnostic radiographs or a significant change in density is noted at the root region, the smart clinician may expect an extra root [5]. Radiographs taken at different horizontal angles can be helpful in detection of the morphology of extra roots [16]. If extra roots are suspected, CBCT is recommended. Prescribed radiographic examinations should be justifiable their advantages should outweigh their health risks [17]. CBCT is a complementary modality for two-dimensional dental imaging [18]. A limited area on a CBCT scan can show an image of several teeth with a radiation dose almost equal to that of two periapical radiographs. This technique can decrease the absorbed dose in patients with complex discrepancies [19]. CBCT can be used in endodontics for diagnostic purposes in difficult cases such as possible accessory canals, root canal anomalies or its curvatures, periapical lesions without clinical symptoms, non-odontogenic pathological lesions with adjacent tissues’ invasion, endodontic treatment complications (overfilling, calcified canals, broken instrument, perforations), root resorption and periradicular surgeries [20, 21]. However, the most definite way for determining root canal morphology is precise observation of the pulp chamber floor. A properly designed access cavity eliminates the majority of problems usually encountered during canal preparation [22]. In this case, an over enlarged access cavity was required to gain straight line access to all four canals (especially those in the M root). In such cases, square- or rectangular-shaped access cavities are preferred over the conventional triangular form for clearer observation of the orifices. In the present case, the pulp chamber floor had a rectangular shape with M canal orifices being distant to each other and the two D canals were closer together.

## Conclusion

The present case report highlights the importance of having comprehensive knowledge about the common and uncommon anatomical forms of the root canals and their possible morphological variations in order to perform satisfactory endodontic treatment.

## References

[1] Christie WH, Peikoff MD, Fogel HM (1991). Maxillary molars with two palatal roots: a retrospective clinical study. J Endod.

[2] Malagnino V, Gallottini L, Passariello P (1997). Some unusual clinical cases on root anatomy of permanent maxillary molars. J Endod.

[3] Siqueira JF Jr (J Endod. 2008). Rocas IN. Clinical implications and microbiology of bacterial persistence after treatment procedures.

[4] Simsek N, Keles A, Bulut ET (2013). Unusual root canal morphology of the maxillary second molar: a case report. Case Rep Dent.

[5] Beatty RG (1984). A five-canal maxillary first molar. J Endod.

[6] Shakouie S, Mokhtari H, Ghasemi N, Gholizadeh S (2013). Two-rooted maxillary first molars with two canals: a case series. Iran Endod J.

[7] Wong M (1991). Maxillary first molar with three palatal canals. J Endod.

[8] Bond JL, Hartwell G, Portell FR (1988). Maxillary first molar with six canals. J Endod.

[9] Holderrieth S, Gernhardt CR (2009). Maxillary molars with morphologic variations of the palatal root canals: a report of four cases. J Endod.

[10] Peikoff MD, Christie WH, Fogel HM (1996). The maxillary second molar: variations in the number of roots and canals. Int Endod J.

[11] Stone LH, Stroner WF (1981). Maxillary molars demonstrating more than one palatal root canal. Oral Surgery, Oral Medicine, Oral Pathology.

[12] Shojaeian S, Ghoddusi J, Hajian S (2013). A case report of maxillary second molar with two palatal root canals and a furcal enamel pearl. Iran Endod J.

[13] Asgary S (2007). Endodontic treatment of a maxillary second molar with developmental anomaly: a case report. Iran Endod J.

[14] Hartwell G, Bellizzi R (1982). Clinical investigation of in vivo endodontically treated mandibular and maxillary molars. J Endod.

[15] Weine FS, Healey HJ, Gerstein H, Evanson L (1969). Canal configuration in the mesiobuccal root of the maxillary first molar and its endodontic significance. Oral Surg Oral Med Oral Pathol.

[16] Jacobsen EL, Nii C (1994). Unusual palatal root canal morphology in maxillary molars. Endod Dent Traumatol.

[17] Gröndahl HG, Huumonen S (2004). Radiographic manifestations of periapical inflammatory lesions. Endodontic topics.

[18] Pinsky H, Dyda S, Pinsky R, Misch K, Sarment D (2014). Accuracy of three-dimensional measurements using cone-beam CT.

[19] Chau A, Fung K (2009). Comparison of radiation dose for implant imaging using conventional spiral tomography, computed tomography, and cone-beam computed tomography. Oral Surgery, Oral Medicine, Oral Pathology, Oral Radiology, and Endodontology.

[20] Cotton TP, Geisler TM, Holden DT, Schwartz SA, Schindler WG (2007). Endodontic applications of cone-beam volumetric tomography. J Endod.

[21] Noujeim M, Prihoda T, Langlais R, Nummikoski P (2009). Evaluation of high-resolution cone beam computed tomography in the detection of simulated interradicular bone lesions. Dentomaxillofac Radiol.

[22] Baratto-Filho F, Fariniuk LF, Ferreira EL, Pecora JD, Cruz-Filho AM, Sousa-Neto MD (2002). Clinical and macroscopic study of maxillary molars with two palatal roots. Int Endod J.

